# Production and Characterization of Medium-Sized and Short Antioxidant Peptides from Soy Flour-Simulated Gastrointestinal Hydrolysate

**DOI:** 10.3390/antiox10050734

**Published:** 2021-05-06

**Authors:** Chiara Cavaliere, Angela Michela Immacolata Montone, Sara Elsa Aita, Rosanna Capparelli, Andrea Cerrato, Paola Cuomo, Aldo Laganà, Carmela Maria Montone, Susy Piovesana, Anna Laura Capriotti

**Affiliations:** 1 Department of Chemistry, Università di Roma “La Sapienza”, Piazzale Aldo Moro 5, 00185 Rome, Italy; chiara.cavaliere@uniroma1.it (C.C.); saraelsa.aita@uniroma1.it (S.E.A.); andrea.cerrato@uniroma1.it (A.C.); aldo.lagana@uniroma1.it (A.L.); susy.piovesana@uniroma1.it (S.P.); annalaura.capriotti@uniroma1.it (A.L.C.); 2 Istituto Zooprofilattico Sperimentale del Mezzogiorno, Via Salute 2, Portici, 80055 Naples, Italy; angela.montone@izsmportici.it; 3 Department of Industrial Engineering, Università degli Studi di Salerno, Via Giovanni Paolo II 132, 84084 Fisciano, Italy; 4 Department of Agriculture Sciences, University of Naples “Federico II”, Via Università 100, Portici, 80055 Naples, Italy; capparel@unina.it (R.C.); paola.cuomo@unina.it (P.C.); 5 CNR NANOTEC, Campus Ecotekne, University of Salento, Via Monteroni, 73100 Lecce, Italy

**Keywords:** antioxidative activity, bioactive peptides, peptidomics, mass spectrometry, soybean

## Abstract

Soybeans (*Glycine max*) are an excellent source of dietary proteins and peptides with potential biological activities, such as antihypertensive, anti-cholesterol, and antioxidant activity; moreover, they could prevent cancer. Also, soy contains all the essential amino acids for nutrition; therefore, it represents an alternative to animal proteins. The goal of this paper was the comprehensive characterization of medium-sized and short peptides (two to four amino acids) obtained from simulated gastrointestinal digestion. Two different analytical approaches were employed for peptide characterization, namely a common peptidomic analysis for medium-sized peptides and a suspect screening analysis for short peptides, employing an inclusion list of exact *m*/*z* values of all possible amino acid combinations. Moreover, fractionation by preparative reversed-phase liquid chromatography was employed to simplify the starting protein hydrolysate. Six fractions were collected and tested for antioxidative activity by an innovative antioxidant assay on human gastric adenocarcinoma AGS cell lines. The two most active fractions (2 and 3) were then characterized by a peptidomic approach and database search, as well as by a suspect screening approach, in order to identify potential antioxidant amino acid sequences. Some of the peptides identified in these two fractions have been already reported in the literature for their antioxidant activity.

## 1. Introduction

In the last few decades, the opportunity to prevent, alleviate, or even treat some diseases by assuming functional food or bioactive compounds obtained from food is gaining increasing interest for both researchers and consumers. Therefore, in this field, efforts are aimed at the discovery of new bioactive compounds and new physiological functions of known compounds, as well as the identification of the most suitable and sustainable foods to extract these valuable substances at the industrial level.

It is known that certain amino acid sequences can have one or more biological functions; for this reason, many studies are focused on the identification of bioactive peptides, which can be naturally present in food or can be obtained from the parent protein in which they are encrypted [1,2]. These bioactive peptides are generally 2–20 amino acids long, even if some bioactivities have been attributed to longer sequences (up to 43 amino acids, as in lunasin).

Bioactive peptides can be released from the parent protein during food processing (such as ripening, fermentation, and cooking), storage, or gastrointestinal digestion [2,3]. For research and industrial applications, several methodological approaches are available to obtain potential bioactive peptides encrypted in proteins, including chemical, physical, and biological ones. Among these, treatment with enzymes is the most suitable one for preserving functional and nutritional values of protein hydrolysate [4]. Nonetheless, the high cost, as well as production of bitter-tasting hydrolysates, are some of the main drawbacks in the employment of enzymes. To better simulate the physiological conditions in which peptides are formed from proteins, human digestive enzymes, which are found in the stomach, intestines, and pancreas (e.g., pepsin, trypsin, chymotrypsin, and pancreatin) can be used [5].

Since dietary proteins represent a cheap and valuable source of bioactive peptides, several foods of both animal and plant origin [2,6,7] have been investigated for this aim, including milk [3,8,9,10], meat, fish [11,12], egg, cereals, and soybean [1,3,7,13].

Soybean (*Glycine max*) has been cultivated for five millennia in Asian countries where, together with its derived products (e.g., soy milk, miso, tofu, etc.), it represents an important source of proteins (ca. 40% of the content) and peptides. Since the last century, soybean cultivation has become widespread in western countries, too [4]. Soy proteins contain all the essential amino acids, and therefore they represent a valid alternative to food of animal origin; furthermore, several biological functions and bioactivities have been attributed to their derived peptides [13], such as cancer and cardiovascular disease prevention, antihypertensive activity [14], hypocholesterolemic effect, and antioxidant properties [1,4,7,15,16]. In particular, the antioxidant activity, i.e., the defense against the free radical damage [17], could be useful in contrasting several pathologies, as well as in nutraceutical and cosmetics applications.

In the present work, we wanted to exploit the antioxidative activity of soybean peptides. Generally, antioxidant sequences are 3–6 amino acid long [6], which are easily metabolized and absorbed in the gastrointestinal tract. Therefore, we focused our attention on short and medium-sized peptides obtained by simulated gastrointestinal digestion of soy protein extracts. First, the soy hydrolysate was separated into six fractions, which were tested for antioxidative activity using an innovative, intracellular reactive oxygen species (ROS) detection assay; in this way, cytotoxic fractions were identified and discarded. Then the peptides, contained in two fractions showing the highest antioxidative properties, were identified using two different strategies for medium-sized and short peptides, respectively. Medium-sized peptides were analyzed by a shotgun proteomic approach, using nano-ultra-high-performance liquid chromatography coupled to tandem mass spectrometry (nanoUHPLC-MS/MS), with identification based on a database search using Proteome Discover software. For short peptides (2–4 amino acid long), the analysis was carried out by UHPLC-MS/MS, following the suspect screening strategy, and their identification was assisted by Compound Discoverer software. Indeed, short peptides have not been fully elucidated in soy yet, mainly because their identification is challenging and cannot be obtained using conventional proteomics approaches and informatics tools. Among the identified peptides, seven were already validated for antioxidative activity and are reported in a public database of food bioactive peptides.

## 2. Materials and Methods

### 2.1. Materials

All chemicals, reagents, and organic solvents of the highest grade available were purchased from Sigma-Aldrich (St. Louis, MO, USA) unless otherwise stated. Trifluoroacetic acid (TFA) was supplied by Romil Ltd. (Cambridge, UK). Ultrapure water was prepared by an Arium 611 VF system from Sartorius (Göttingen, Germany). Mass grade solvents used for medium-sized peptides were purchased from VWR International (Milan, Italy). Optima LC-MS grade water and acetonitrile (ACN), used for short peptide analysis, were purchased from Thermo Fisher Scientific (Waltham, MA, USA).

### 2.2. Protein Extraction

Soy flour samples were purchased in a local market. An aliquot of 2.7 g of soy flour was extracted with 15 mL of a cold buffer consisting of 6 mol L^−1^ urea, 10 mmol L^−1^ Tris (hydroxymethyl) aminomethane hydrochloride (Tris-HCl, pH 8), 75 mmol L^−1^ NaCl. The sample was vortexed for 50 min, then centrifuged at 9400× *g* at 4 °C for 30 min. All protein samples were quantified by the bicinchoninic acid (BCA) assay using bovine serum albumin (BSA) as standard, and stored at −80 °C until digestion. 

#### Protein Digestion

A simulated in vitro gastrointestinal digestion was performed as previously described [18], with some modifications. The pH of the protein extract was adjusted to 2.0 with 1 mol L^−1^ HCl to mimic the stomach environment, then pepsin was used in an enzyme-to-protein ratio of 1:20. The solution was incubated at 37 °C for 1 h under static conditions. Then, the pH was adjusted to 7.5 with 1 mol L^−1^ NaOH to stop pepsin digestion and create the best environment for pancreatin hydrolysis; pancreatin was added with an enzyme-to-protein ratio of 1:10. After 2 h, α-chymotrypsin was added with an enzyme-to-protein ratio of 1:20. The solution was incubated overnight at 37 °C. Enzymatic hydrolysis was stopped by decreasing the pH to 2.0 with TFA. The volume of the digested extract was reduced to 30 µL by an IKA RV 8 rotary evaporator (IKA-Werke GmbH & Co. KG, Staufen, Germany) for subsequent analyses.

### 2.3. Purification of Antioxidant Peptides from Soybeans

The hydrolyzed peptides were purified by preparative liquid chromatography (LC) using the Xbridge BEH C18 OBD Prep, 19 mm id × 250 mm column (particle size = 5 μm; Waters, Milford, MA, USA) as previously described [19,20], with some modifications. The chromatographic system was a Shimadzu Prominence LC-20A, including a CBM-20A controller, two LC-20 AP preparative pumps, and a DGU-20A3R inline degasser. An SPD-20A UV detector with a preparative cell (0.5 mm) was used. The FRC-10A (Shimadzu, Kyoto, Japan) auto-collector was employed. Data acquisition was performed by LabSolution software version 5.53 (Shimadzu, Kyoto, Japan). The detector was set at 214 nm.

**Figure 1:**
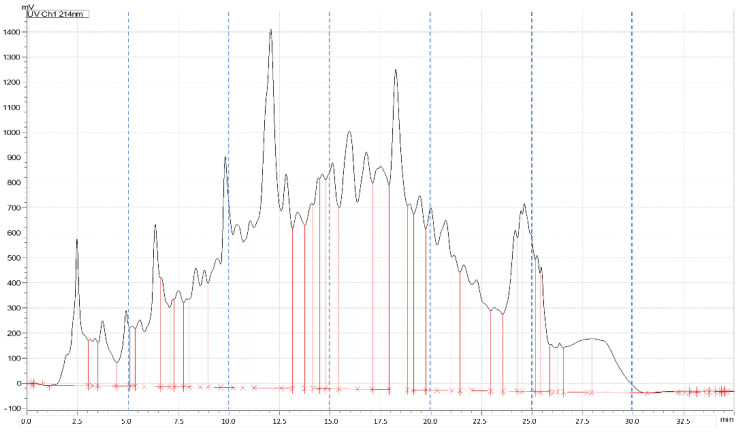
LC-UV chromatogram of hydrolyzed soybean extracts showing the applied fractionation.

Chromatography was operated at a flow-rate of 17 mL min^−1^_,_ using H_2_O with 10 mmol L^−1^ ammonium formate at pH 10 as phase A, and methanol/H_2_O (90/10, *v*/*v*, with 10 mmol L^−1^ ammonium formate, pH 10) as phase B. The chromatographic gradient was as follows: B was increased from 0% to 50% in 20 min, then B was brought to 95% in 4 min and kept constant for 6 min. The column was equilibrated at starting condition for 6 min. Six fractions were collected every 5 min (as shown in Figure 1). Each peptide fraction was evaporated with a rotary evaporator and stored at −80 °C for subsequent analysis.

### 2.4. Bioactivity Assays

#### 2.4.1. Cell Culture

Human gastric adenocarcinoma cell line AGS (ATCC CRL-1739) was grown in DMEM-F12 medium, supplemented with 10% fetal bovine serum (FBS), 1% glutamine, and 1% penicillin + streptomycin (all from Microtech, Vero Beach, FL, USA) and cultured in T-75 flasks in a humidified incubator containing 5% CO_2_ at 37 °C.

#### 2.4.2. Intracellular Reactive Oxygen Species Measurement

AGS cells were split at 80–90% of confluency, seeded (0.5 × 10^6^) in 35 mm culture dishes, and incubated at 37 °C in a 5% CO_2_ atmosphere overnight. After cell attachment, ROS detection assay was assayed using dihydrorhodamine 123 (DHR, Sigma Aldrich, St. Louis, MO, USA), as described by Cuomo et al. [21]. Briefly, cells were loaded with DHR (10 μmol L^−1^ for 20 min), and were treated with (1) 1 mmol L^−1^ H_2_O_2_ for 30 min; (2) 1 mg mL^−1^ soybean peptide fractions for 1 h, and (3) 1 mg mL^−1^ soybean peptide fractions for 1 h and 1 mmol L^−1^ H_2_O_2_ for 30 min. After treatments, DAPI (Thermo Fischer Scientific, Waltham, MA, USA) was used as a nuclear counterstain, and cells were analyzed with a Zeiss Axioskop 2 Hal100 fluorescence microscope equipped with a digital camera (Nikon, Tokyo, Japan). The excitation and emission wavelengths were 488 and 515 nm, respectively. Images were digitally acquired with exposure times of 100–400 ms, and processed for fluorescence determination with ImageJ software. The fluorescence signal of the intracellular ROS was normalized on the DAPI fluorescence signal.

### 2.5. Analysis of Medium-Sized Peptides by nanoUHPLC-MS/MS

The chromatographic system was an Ultimate 3000 nanoUHPLC (Thermo Scientific, Bremen, Germany) coupled to an Orbitrap Elite mass spectrometer (Thermo Scientific). Pierce LTQ Velos ESI Positive Ion Calibration Solution (Thermo Fisher Scientific) was used to calibrate the instrument once a week, and the mass accuracy was <1.5 ppm.

Medium-sized peptides were analyzed as described in a previous work [22], with some modifications. A 10 µL sample was injected and preconcentrated on a μ-precolumn (300 μm id × 5 mm, Acclaim PepMap 100 C18, particle size 5 μm, pore size 100 Å; Thermo Fisher Scientific) at a 10 μL min^−1^ flow-rate of H_2_O/ACN 99:1 (*v*/*v*) containing 0.1% (*v*/*v*) TFA. Then the peptide mixture was separated on an EASY-Spray column (Thermo Fisher Scientific, 75 μm id × 15 cm, PepMap C18, 3 μm particles, 100 Å pore size) operated at 300 nL min^−1^ and 35 °C.

The mobile phase was constituted by (1) H_2_O and (2) ACN, both with 0.1% formic acid. The chromatographic gradient, referred to as B, was 1% for 5 min, 1–5% in 2 min, 5–35% in 90 min, and 35–90% in 3 min. Finally, the column was rinsed at 90% B for 10 min and equilibrated at 1% B for 19 min.

Full-scan mass spectra were acquired in the 300–2000 *m*/*z* range at 30,000 resolution (full width at half maximum, FWHM, at *m*/*z* 400). Tandem mass spectra were acquired at 15,000 resolution (FWHM, at *m*/*z* 400) in the top 10 data-dependent acquisition (DDA) mode, with the rejection of singly charged ions and unassigned charge states. Precursor ions were fragmented by higher-energy collisional dissociation (HCD), with 35% normalized collision energy (NCE) and a 2 *m*/*z* isolation window. Dynamic exclusion was enabled with a repeat count of 1 and a repeat duration of 30 s, with an exclusion duration of 20 s. For each sample, three technical replicates were performed. Raw data files were acquired by Xcalibur software (version 2.2, Thermo Fisher Scientific).

### 2.6. Analysis of Short Peptides by UHPLC-MS/MS

The short peptides were analyzed by reverse-phase (RP) chromatography, using the Vanquish H UHPLC system coupled to an Q Exactive mass spectrometer (Thermo Fisher Scientific, Bremen, Germany) and a heated electrospray source (ESI) [23]. The instrument was calibrated every 48 h using a Pierce LTQ Velos ESI Positive Ion Calibration Solution (Thermo Fisher Scientific); mass accuracy was <1 ppm. The peptide mixtures were separated on a Kinetex XB-C18 chromatographic column (2.1 mm id × 100 mm, particle size 2.6 μm; Phenomenex, Torrance, CA, USA) kept at 40 °C. The mobile phase consisted of (1) H_2_O and (2) ACN, both containing 0.1% TFA (*v*/*v*); the flow-rate was 0.4 mL min^−1^. The gradient, referred to as B, was 1% for 2 min, 1–35% in 20 min, and 35–99% in 3 min. Finally, B was maintained at 99% for 3 min and then lowered again to 1%. The MS acquisition was performed in positive ionization mode in the range *m*/*z* 150–750 with a resolution (FWHM, *m*/*z* 200) of 70,000, while MS/MS spectra were recorded in top 5 DDA mode, using 35% NCE for HCD and a resolution of 35,000 (FWHM, *m*/*z* 200). For the DDA (4980 unique masses), an inclusion list with exact *m*/*z* values for individually charged precursor ions was used. The inclusion lists were prepared using MatLab R2018, as previously described [24].

For each sample, three technical replicates were carried out. Raw data files were acquired by Xcalibur software (version 2.2 SP1.48; Thermo Fisher Scientific).

### 2.7. Peptide Identification

#### 2.7.1. Medium-Sized Peptide Identification

The raw files of the digested fractions were submitted to the Proteome Discoverer software (version 1.3; Thermo Scientific) with the Mascot search engine (v.2.3; Matrix Science, London, UK) for the identification of peptides/proteins.

The research was performed against the proteome of *Glycine max* (Soybean) downloaded from Uniprot (http://www.uniprot.org/ accessed on 11 January 2020, 85022 sequences). Nonspecific digestion was chosen, and neither fixed nor variable changes were set. The mass tolerances for the precursor and the product ions were set at 10 ppm and 0.05 Da, respectively. A decoy function was used for false discovery rate calculation, which was set at 1%.

#### 2.7.2. Short Peptide Identification

The identification of short peptides in fractionated gastrointestinal digests was performed following a data processing workflow implemented on Compound Discoverer (v. 3.1, Thermo Fisher Scientific, Bremen, Germany) [25]. The masses were extracted from the raw files based on customized parameters, aligning the signals, removing empty or missing MS/MS spectrum signals, and using short lists of complete peptides to match a possible composition. Manual validation of MS/MS spectra was also aided by the Compound Class Assessment Tool, which allowed to automatically match typical product ions from amino acids at the N-terminus, C-terminus, and in the middle of the sequence, and assign them to 20 classes of compounds (one for each natural amino acid). Provisional identification of short peptides was obtained according to diagnostic fragmentation spectra, aided by mMass, which allows for in silico fragmentation of peptides [26].

## 3. Results and Discussion

### 3.1. Sample Preparation

#### 3.1.1. Extraction

Three different extraction protocols were tested in terms of protein recovery, also aiming to minimize interference content in the extract. Furthermore, the total time required for sample preparation was also considered for a possible industrial scale-up of the process. Experimental details of protocols I and II are reported in Appendix A, whereas protocol III was selected for this work, and is reported in the experimental section. Briefly, protocol I [27] was based on the use of an extraction buffer containing sodium dodecyl sulfate (SDS), whereas glass beads and a buffer containing sodium deoxycholate (SDC) were used in protocol II [28] to enhance the lysis of cell walls. For protocol III, a buffer containing urea was used.

**Figure 2:**
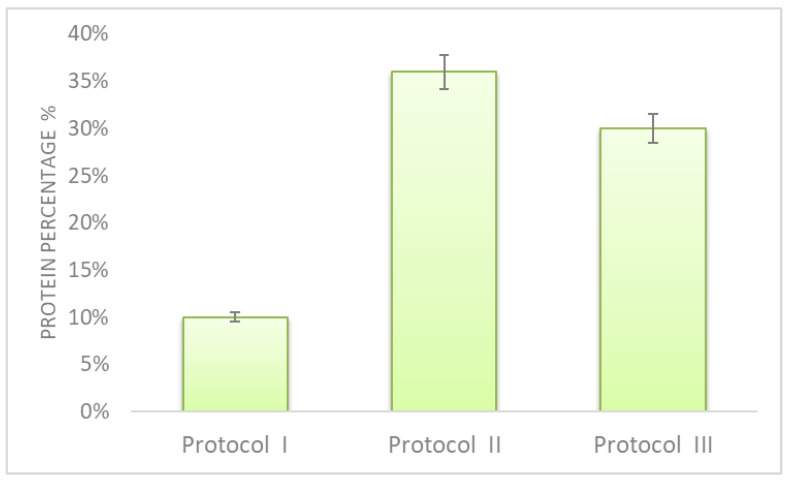
Protein recovery percentage obtained by applying three different extraction protocols. Evaluation was carried out by BCA assay, using BSA as a standard.

A BCA assay was employed to evaluate the protein extraction recovery of the three protocols, and results are shown in Figure 2. Protocol I gave the worst extraction efficiency, and was the most time-consuming; therefore, it is not suitable for a possible industrial scale-up. Protocol II gave the best result; however, the employment of glass beads does not allow us to extract large flour samples. Protocol III provided similar results and was the fastest, and for these reasons was selected.

In protocol III, the employment of urea allows us to not only denature proteins but also to solubilize and extract them, as urea is a mild chaotropic agent. Moreover, all the other buffer constituents were compatible with the following analysis steps.

#### 3.1.2. Gastrointestinal Digestion

In the gut, endogenous proteases, such as pepsin, trypsin, and chymotrypsin, hydrolyze proteins into peptides, which will be further processed by peptidases in the intestinal tract [29]. Therefore, to reproduce gastrointestinal digestion, a sequence of different enzymes is generally used, mainly pepsin, trypsin, chymotrypsin, and papain [17]. 

To obtain putative bioactive peptides from soy by simulated gastrointestinal digestion, different enzymes were employed, mainly alcalase [16,30,31]; however, other proteases, also in combination, were used as well, including bromelain, papain, pepsin, flavourzyme, neutrase, protemax, and transglutaminase [4]. 

In this work, we sequentially used pepsin, papain, and chymotrypsin. During hydrolysis, temperature, pH, and enzyme-to-substrate ratio were carefully checked to obtain reproducible results (data not shown).

### 3.2. Soybean-Derived, Hydrolyzed Peptides in Oxidative Stress In Vitro

Recently, the antioxidant role of soybean, and specifically of peptides derived from hydrolysed soybean proteins, has been reported [15,32,33]. ROS are molecules physiologically produced during cell metabolism, participating in cell proliferation and survival. Nevertheless, external stimuli, i.e., environmental factors, xenobiotics, and microbial infection, may contribute to ROS accumulation, causing an imbalance between their production and removal by cellular antioxidant systems [34]. This can result in oxidative stress, leading to cell damage.

**Figure 3:**
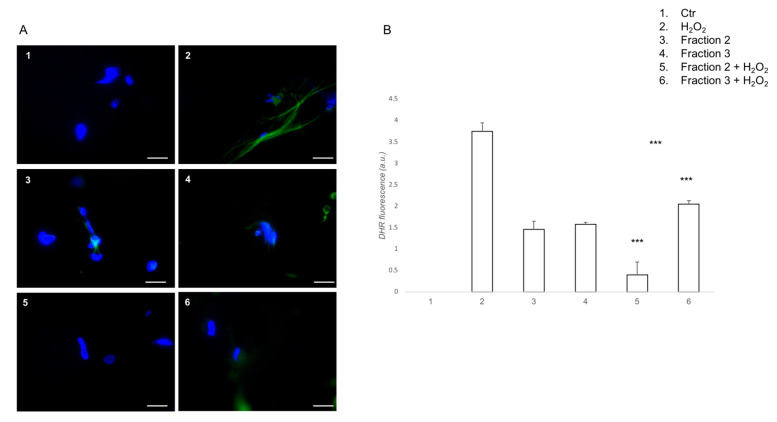
Soybean peptides prevents H_2_O_2_-induced, intracellular ROS formation. (**A**) DHR-loaded cells were pre-treated for 1 h with peptide fractions 2 or 3 (1 mg mL^−1^), then incubated for further 30 min with H_2_O_2_ (1 mmol L^−1^), and finally observed and analyzed with a fluorescence microscope (scale bar: 100 µm); (**B**) Quantification of the mean fluorescence of individual cells. Results are expressed as arbitrary units and represent the average ± SD calculated from three independent experiments, each performed in duplicate. Statistical analysis was performed with ANOVA followed by Bonferroni’s test. *** *p* < 0.001, comparing 5 with 2 or 6 and 6 with 2.

Using a DHR cell-permeable fluorogenic probe, which is useful to detect ROS production, the ability of bioactive soybean peptides was investigated to prevent ROS formation in H_2_O_2_ stimulated cells. Among the six peptide fractions, we tested the antioxidant activity of fractions 2 and 3. The remaining fractions were excluded because of their cytotoxic effect (data not shown). Indeed, the choice of using a gastric cell line was determined by the need for evaluating the potential cytotoxic effect of the derived peptide fractions, justifying its potential use as functional food. As shown in Figure 3, the cells pre-treated with both fractions 2 and 3 displayed reduced ROS levels compared to H_2_O_2_-treated cells. Interestingly, compared to fraction 3, fraction 2 exhibited a higher protective effect against oxidative stress induced by H_2_O_2_, almost returning the cells to the control group. These results suggest that soybean peptides have important antioxidant activities, with particular attention to peptides contained in fraction 2. 

Compared to classical assays used to evaluate the antioxidant activity of soy hydrolysates, such as 2,2-diphenyl-1-picrylhydrazyl (DPPH) [16], oxygen radical absorbance capacity (ORAC) [31], lipid peroxidation inhibitory activity [30], and the employment of an intracellular ROS measurement allowed also to recognize the cytotoxic effect of some fractions.

### 3.3. Identification of Peptides in Fractions with High Antioxidative Activity

The antioxidant function of soy peptides has been attributed to multiple mechanisms, including hydrogen donation, scavenging of hydroxyl radicals, transition-metal ion chelation, and active-oxygen quenching; therefore, it is widely accepted that there is a synergistic effect [4]. Nonetheless, it is still interesting to characterize the most bioactive species, also to better investigate the amino acid sequences responsible for the antioxidative activity. Therefore, the peptides contained in the two most active fractions, namely 2 and 3, were fully characterized and searched in BIOPEP-UMW database for confirmation. 

#### 3.3.1. Analysis of Short Peptides

The identification of short peptides is challenging for several reasons, as already reported [24]. Briefly, the main issues consist of the low possibility of identifying short sequences (<5 amino acids) by a proteomic database search [35], also due to the high occurrence of isobaric peptides and the scarce ionization efficiency of short peptides, which mainly form monoprotonated molecules in positive ESI. Moreover, scarce fragmentation data can be obtained. Therefore, a previously developed, specific method was employed for short peptide identification. Following a HRMS-based suspect screening approach, a list containing the exact masses of precursors relative to all the possible combinations of the 20 natural amino acids (from two to four, resulting in 168,400 unique combinations) was used. 

A total of 132 unique amino acid sequences were identified in fractions 2 and 3. However, under the operating conditions, it was not possible to discriminate the isobaric leucine and isoleucine by tandem mass spectra; therefore, the occurrence of the two amino acids within the identified sequence was retained as equally probable (see Table S1 of Supplementary Materials). Consequently, 203 possible sequences were searched in BIOPEP-UWM database [36], where, on a total of 4216 food bioactive peptides, there are 689 validated sequences for antioxidative activity.

Table 1 reports the short peptides identified in fractions 2 and 3 of the soybean hydrolysate and matching antioxidative sequences reported in BIOPEP-UWM database. The sequences IR and LK, obtained from ovotransferrin protein, showed radical-scavenging activity by ORAC assay [37]. The dipeptide AW was obtained from the marine bivalve *Mactra veneriformis*, and it showed hydroxyl, DPPH, and superoxide radical scavenging activities [38]. The dipeptide EL from casein positively responded to SOSA and DPPH assays [39]. The two last sequences, LH and ADF, were identified in soybean [40] and one of its derived products [41], respectively, and both were tested for antioxidative activity against the peroxidation of linoleic acid.

Most of the other identified di-, tri-, and tetrapeptides are reported in the BIOPEP-UWM database with some biological functions, and the dipeptidyl peptidase IV inhibitor and ACE inhibitor are the most frequently occurring.

The list of the identified short sequences was also submitted to PeptideRanker [42], a server for the prediction of bioactive peptides based on a novel N-to-1 neural network, which assigns a generic bioactivity probability rank. Although 0.5 is the threshold value for labeling a peptide as bioactive, a 0.8 threshold is suggested to reduce false-positive predictions. The rank assignment of the 203 short unique amino acid sequences is reported in Table S2 of the Supplementary Materials: 32 and 15 short peptides had a rank above 0.5 and 0.8, respectively. The latter included 10 dipeptides, 3 tripeptides, and 2 tetrapeptides. Of the six antioxidant peptides found in BIOPEP-UWM, only AW and ADF had a high rank, namely 0.9669 and 0.8062, respectively.

Concerning the amino acid composition of bioactive peptides, the presence of hydrophobic amino acid residues, such as W, F, P, G, K, I, and V, at both the N-terminus and C-terminus, are relevant for peptide antioxidative activity, as well as H and R at the C-terminus position [43]. In particular, the presence of H has been associated with metal-ion chelator, active-oxygen quencher, and hydroxyl radical scavenger properties [4,7]. Another contribution to the antioxidative activity (by free radical quenching) is provided by the excess electrons of the negatively charged residues E and D. Also, the relative positions of these amino acids can determine or enhance their activity [44]. 

All the bioactive short peptides reported in Table 1 present at least one of these characteristic residues. Moreover, all the identified sequences of our soybean hydrolysate with a prediction rank >0.8 assigned by PeptideRanker contain at least one of these amino acids. 

#### 3.3.2. Analysis of Medium-Sized Peptides

As expected, most of the medium-sized peptides are derived from glycinin, which is the major seed storage protein of soybean, β-conglycinin, and their isoforms and subunits. Indeed, these two proteins account for up to 80–90% of the total soy protein content. The peptide identification in fractions 2 and 3 are reported in Tables S3 and S4 of the Supplementary Materials, respectively. 

Table 2 shows the only medium-sized peptide, identified in fraction 3, that matched with a validated antioxidant sequence in the BIOPEP-UWM database. 

PeptideRanker was used to predict the general bioactivity of the medium-sized peptides as well. The peptide reported in Table 2 obtained a very low prediction score. In general, among the 136 sequences identified in fraction 2, 33 and 19 peptides had bioactivity prediction scores >0.5 and >0.8, respectively (see Table S5 in Supplementary Materials). Concerning fraction 3, only 3 and 1 sequences out of a total of 66 presented a score >0.5 and >0.8, respectively (see Table S6 of Supplementary Materials).

#### 3.3.3. Further Considerations on Identified Peptides

**Figure 4:**
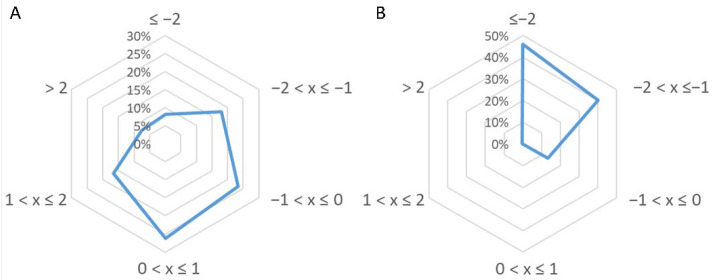
Gravy index score distribution for (**A**) short peptides and (**B**) medium-sized peptides identified in fractions 2 and 3 of soybean hydrolysate.

The characteristics of peptides identified in the two active fractions were further analyzed by their grand average of hydropathy (GRAVY) index score, which is usually used to estimate the hydrophobicity or hydrophilicity of a protein. The resulting index, ranging between −2 and +2, will be positive for hydrophobic amino acid sequences. Figure 4A,B reports the GRAVY value distributions for short and medium-sized peptides. As is known, medium-sized peptides are more hydrophilic than short peptides.

Generally, antioxidant peptides, besides containing the amino acids reported above, have a low molecular mass and a GRAVY score ranging between −0.5 and +0.5 [45]. About 50% of the short peptides identified in our research also meet the last criterion, as can be seen in Figure 4A. Regarding medium-size peptides, this requisite is possessed only by a small percentage (Figure 4B).

## 4. Conclusions

In this work, peptide fractions obtained by the simulated gastrointestinal digestion of soybean protein extracts were evaluated for antioxidative activity by an intracellular test. Only the most active and non-cytotoxic fractions were fully characterized for their short and medium-sized peptide content. The analysis of the two peptide typologies required two different analytical approaches and two diverse software packages for their identification. In particular, the challenge in the identification of short peptides is the impossibility of following the classical informatics tools generally employed in proteomics analysis.

Among the various peptide sequences identified, five dipeptides, one tripeptide, and one medium-sized peptide had already been reported to possess antioxidative activity in the most up-to-date database of food bioactive peptides. In particular, one dipeptide, the tripeptide, and the medium-sized peptide were already identified in soy or a soy by-product. A further purification of the two active fractions (containing almost the same peptides) could help in identifying the peptides with the highest antioxidant function. This could be particularly interesting for the short peptides, which are less frequently investigated for the known issues related to their analysis, and which have not been fully elucidated in soy yet. Nevertheless, it is important to keep in mind that the amino acid sequences could exert their bioactivity only in combination or with a synergic effect. Furthermore, the short peptides could be tested for assessing other bioactivities.

Nowadays, soy cultivation is widespread in several countries, mainly for feed production. Nonetheless, the economic benefits derived from its intensive cultivation are controversial, because of the negative ecological impact on soil, forests, and other plant cultivation. For this reason, the possibility of maximizing the exploitation of this plant and its by-products by extracting the most active amino acid sequences for several industrial applications, including nutraceuticals, could support its sustainable employment.

## Figures and Tables

**Table 1:** Short peptides matching with BIOPEP-UWM sequences validated for antioxidative activity. Peak areas are average values of all the replicates.

Sequence	Peak Area Fraction 2 (×10^6^)	Peak Area Fraction 3 (×10^6^)	Peptide Source
Ile–Arg (IR)	13	815	Egg white ovotransferrin [37]
Leu–Lys (LK)	692	0.4	Egg white ovotransferrin [37]
Ala–Trp (AW)	1	332	Marine bivalve [38]
Glu–Leu (EL)	261	2	Milk casein [39]
Leu–His (LH)	2	92	Soybean [40]
Ala–Asp–Phe (ADF)	124	0.9	Okara [41]

**Table 2:** Medium-sized peptide identified in fraction 3 that matched with a BIOPEP-UWM sequence validated for antioxidative activity.

Sequence	Peptide Source	PeptideRanker Score
VNPESQQGSPR	Soy [17]	0.155249

## Data Availability

Data is contained within the article.

## Supplementary Materials

The following are available online at https://www.mdpi.com/article/10.3390/antiox10050734/s1, Table S1: Short peptide identification, Table S2: Short peptide bioactivity prediction by PeptideRanker, Table S3: Medium-sized peptides identified in fraction 2, Table S4: Medium-sized peptides identified in fraction 3, Table S5: Medium-sized peptide (fraction 2) bioactivity prediction by PeptideRanker, Table S6: Medium-sized peptides (fraction 3) bioactivity prediction by PeptideRanker.

## Appendix A. Comparison of Three Protein Extraction Protocols

Protocol I: soy flour (2.7 g) was added to 1 mL of extraction buffer, i.e., 50 mmol L^−1^ Tris (pH 8.8), 15 mmol L^−1^ KCl, 20 mmol L^−1^ dithiothreitol (DTT), and 1% (*w*/*v*) sodium dodecyl sulfate (SDS) [27]. Then, the sample was placed on ice for 1 h, and vortexed for 1 min every 15 min. Insoluble matter was removed by centrifugation at 4 °C for 15 min at 11,000× *g*. The supernatant was collected, and the proteins were precipitated overnight at −20 °C by adding four volumes of acetone. After centrifugation at 11,000× *g* at 4 °C for 30 min, the pellet was dried and dissolved in 6 mol L^−1^ of urea and 10 mmol L^−1^ Tris-HCl (pH 8).

Protocol II: proteins were extracted from soy flour with the aid of glass beads [28]. A 25 mg soy sample was added to 150 mg glass microbeads and 1 mL lysis buffer (60 mmol L^−1^ Tris pH 9, 15 mmol L^−1^ KCl, 20 mmol L^−1^ DTT, 2% (*w*/*v*) SDC). The sample was placed in an ultrasonic bath for 4 h, under agitation for 1 h every 30 min. Then, the sample was incubated at 100 °C for 30 min; centrifugation at 4 °C at 11,000× *g* for 15 min allowed us to remove insoluble matter. The supernatant was collected as proteins precipitated, as described above.

Protocol III: this is reported in the experimental section, and was used for this work.
