# Supplementary material for: Production and Characterization of Medium-Sized and Short Antioxidant Peptides from Soy Flour-Simulated Gastrointestinal Hydrolysate
Source: Antioxidants (Basel). 2021 May 6;10(5):734. doi: 10.3390/antiox10050734 (PMC8148578; doi:10.3390/antiox10050734)
Supplement: Supplementary file 1 [file antioxidants-10-00734-s001.zip › antioxidants-1188856-supplementary.pdf]

Table S6. MEDIUM-SIZED PEPTIDES (FRACTION 3) BIOACTIVITY PREDICTION BY PEPTIDERANKER

rank	sequence
0.829165	WQEQQDEDEDEDEDDEDEQIPSHPPRRPSHG
0.78413	WQEQQDEDEDEDEDDEDEQIPSHPPRRPS
0.597538	HGGKGSEEEQDEREHPRPHQPH
0.475004	QNQLDQMPR
0.459632	SGFEGDNMIER
0.395137	KPEDWDDKEFIPDPEDKKPEG
0.351856	ENQLDQMPR
0.323857	HDKSSPSYHR
0.316052	KSQSDNFEYV
0.273056	PRPPHQKEER
0.270425	SAMPGHGTGQPTGH
0.257461	SPQLENLRD
0.257193	NALEPDHR
0.249465	EEFFGPGGRDPESV
0.245269	NNQLDQNPR
0.239882	HFNEGDVL
0.234141	NALKPDNR
0.226605	DRIYDYDV
0.222875	NNQLDQTPR
0.210959	HDKSSPSYH
0.190933	SEFEEINRV
0.182809	GKGGGIEVDSTGK
0.172989	KTNDRPSIGN
0.171477	KAADQIAGQT
0.171255	MELENLEERW
0.167042	RPSYTNGPQEI
0.156414	KTISSEDEPF
0.155249	VNPESQQGSPR
0.144776	ERIYDYDV
0.142142	SVVGGDGLPDTVEK
0.135155	GINAENNQRN
0.133299	NHGDQILDEANKA
0.127302	SYDTKFEEIN
0.124885	STGHVPSSGGESSA
0.121697	VVAEQAGEQGFE
0.119904	LEHGGIATDDDYP
0.115885	YGTNTADTGTGPR
0.115207	LAASAGETAKE
0.109199	RQNIGQNSSPDI
0.108575	RAELSEDDVFV
0.101717	NEGEANIEL
0.0960369	KLQGENEEEEKGAIVT
0.0960217	TSVGHMPSTKEEGH
0.0936312	GHGHHTTGTGTGTAT
0.09185	KESDITAGKDTPQGSIEA

0.0897102	KEDVKVEVEDGRV
0.0875467	EKLQDIASEAGQ
0.0854157	GLVSESETEKI
0.0849549	DLTEDDLIEK
0.0847615	IKETVVGKDDDDDDDHGDGH
0.0838137	NTNEDIAEKL
0.0837387	HNIGQTSSPD
0.0809551	GEEEEQRQQEGVIVEL
0.0798314	DTKFEEIN
0.079601	KIEAEGTQSGTPK
0.0770362	RHNIGQTSSPD
0.0763415	EITPEKNPQ
0.072587	RNLQGENEEEDSGAIV
0.0713902	KHHDVTVKDEK
0.0699062	KVDEYGNVEK
0.0659821	KEEVKVEVEDGRV
0.0525346	QKIMENQSEELEEK
0.0518491	TLVNNDDRDS
0.0453637	KIMENQSEELEEK
0.0443023	QKIMENQSEELEEKQ
0.0441246	IMENQSEELEEK
